# Examining the coupling relationship between industrial upgrading and eco-environmental system in resource-based cities in China

**DOI:** 10.3389/fpubh.2025.1527306

**Published:** 2025-02-14

**Authors:** Yu Lei, Yongxiang Chen, Lanyue Zhang, Yifu Lu

**Affiliations:** ^1^Chengdu Jincheng College, Chengdu, China; ^2^Chengdu University of Technology, Chengdu, China; ^3^Sichuan University Jinjiang College, Meishan, China; ^4^Australian National University, Canberra, ACT, Australia

**Keywords:** industrial structure upgrading, ecological environment, entropy-CRITIC-TOPSIS model, coupling coordination model, resource-based city

## Abstract

**Introduction:**

The long-term extensive development of resource-based cities makes them face problems such as resource exhaustion, industrial structure imbalance, ecological environment deterioration, which urgently need to carry out green transformation.

**Methods:**

In this study, Entropy-CRITIC-TOPSIS model and coupling coordination degree model were constructed to estimate and analyze the coordinated development level of industrial structure upgrading and ecological environment of 19 resource-based cities in Northeast China from 2010 to 2021.

**Results:**

The results showed that the coupling coordination situation between industrial structure rationalization and ecological environment in 19 resource-based cities were unsatisfactory, and the proportion of uncoordinated development between industrial structure rationalization and ecological environment was as high as 89.47%. In comparison, the coupling coordination situation between industrial structure advancement and ecological environment were relatively better, with all cities’ industrial structure upgrading and ecological environment systems being basically coordinated or above.

**Discussion:**

This suggests that resource-based cities in Northeast China should still focus on improving the rational allocation of resources, environmental protection, and coordinated development of resource utilization. The conclusion of this study provides a theoretical basis for the sustainable development of domestic resource-based cities, and can also be used as a reference for domestic and foreign resource-based cities or regions.

## 1 Introduction

Since the reform and opening up in 1978, China has accelerated the pace of industrialization, achieved sustained and rapid economic development, and made remarkable contributions to the world. However, the traditional industrial development model, driven by economic interests, neglects the protection of resources and the environment (1). This has led to a series of problems, including excessive resource consumption, high carbon emissions (2), and worsening air pollution, such as haze, which not only threatens public health but also results in significant economic losses (3). How to balance the relationship between economic development and ecological environment (EE) is a problem that China and most countries and regions need to solve urgently to implement sustainable economic development. China has always firmly established the concept that clear waters and green mountains are as valuable as mountains of gold and silver, and integrated the requirements of green transformation into the overall economic and social development. Industrial structure, as a key component of the economic system, is crucial for achieving sustainable development in a city or region. An unreasonable industrial structure disrupts the balanced development of the economy and leads to a focus on traditional, low-level development models, resulting in environmental pollution (4, 5). The secondary industry will significantly increase energy consumption and environmental pollution, while the tertiary industry emits relatively little pollution (6), which may be due to the fact that the secondary industry contains more pollution-intensive enterprises, and this phenomenon is more obvious in resource-based cities.

Resource-based cities are formed and developed through the exploitation and processing of mineral resources, forests, and other natural resources, and their development model is closely tied to the abundance of these resources (4, 5, 7). As an important part of economic development, resource-based cities have long provided crucial resources and energy for China’s industrial transition and economic growth, making significant contributions to the establishment of an independent and complete industrial system and the promotion of national economic development. However, the long-term extensive development of resource-based cities makes them face multiple problems such as resource exhaustion, unbalanced industrial structure, unsatisfactory social welfare, environmental pollution, land degradation and population loss (8), which affects the healthy development of economy, society and environment. In recent years, resource-based cities not only have prominent environmental problems and unbalanced regional industrial structure, but also gradually lose their investment attractiveness, which hinders the further development of economy and is contrary to the sustainable development strategy advocated by the state (9–11).

**Figure 1:**
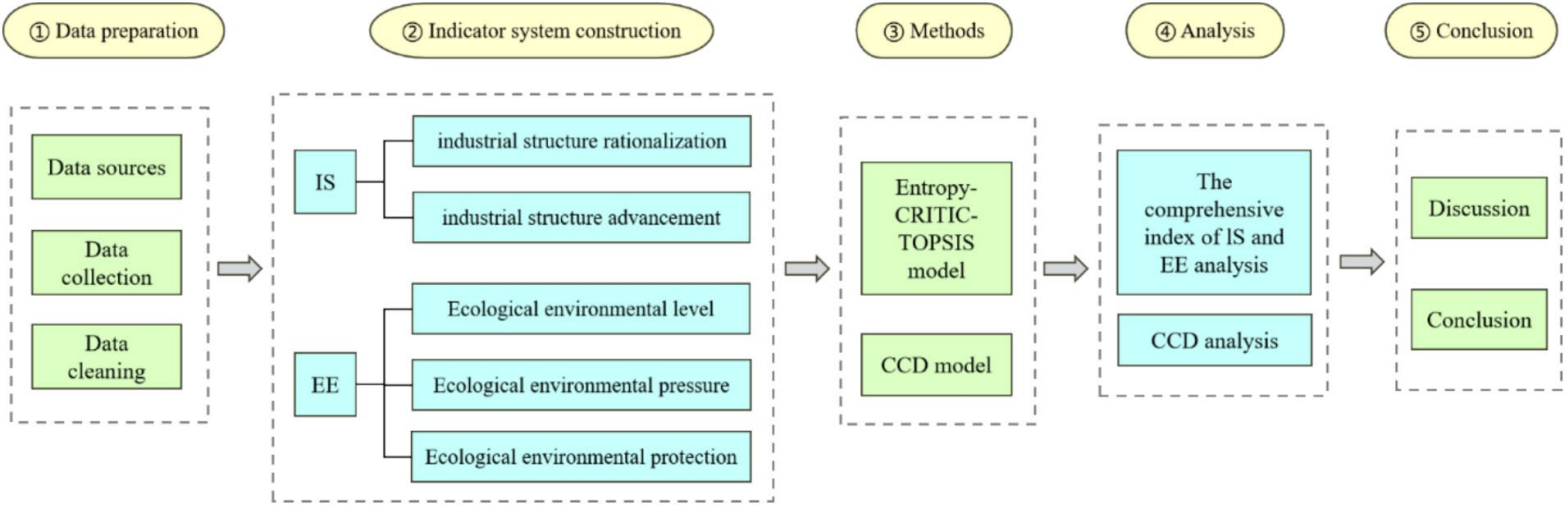
The theoretical analysis framework.

The industrial structure upgrading, improvement of ecological environment and sustainable urban development have become the focus of the transformation of resource-based cities. The Chinese government has adopted a series of measures, including green technology innovation, industrial structure upgrading and environmental regulation of industrial enterprises to promote the industrial transformation development from extensive to intensive (3, 6, 12, 13). The upgrading of industrial structure can improve carbon emission performance (14), and ecological environmental protection and environmental regulations can stimulate enterprises to engage in green technological innovation, further amplifying the positive impact on the upgrading of industrial structure through economic development and resource endowments (15). However, in the process of the development of resource-based cities, the resources which they rely on for survival will gradually be exhausted (16). If the industrial development mode is not changed, this resource advantage will eventually be transformed into a “resource curse.” Therefore, it is one of the major strategic issues facing China to promote industrial structure upgrading of resource-based cities, improve their ecological environment and achieve sustainable urban development. This study will focus on the coordinated development of industrial structure upgrading and ecological environment system of resource-based cities and their mutual influence, which is an important research direction under the background of national sustainable development strategy. By constructing the coupling coordination degree model of industrial structure upgrading and ecological environment system of 19 resource-based cities in Northeast China, this study calculated the coupling coordination degree (CCD) of these two subsystems, and then deduced the relevant conclusions. The theoretical analysis framework is shown in Figure 1.

## 2 Literature review

Industrial structure is the link between industrial activities and energy efficiency, and determines the allocation tendency and output efficiency of production factors to a certain extent (17). The industrial structure upgrading refers to the process of transforming the industrial structure from a low level to a high level (18, 19). It also involves continuously optimizing resource allocation within industries through persistent coordination of both internal conditions and the external environment of industrial development, thereby driving significant improvements in industrial efficiency (20). The industrial structure of areas with low technical level and rich natural resources and labor resources belongs to the lower level, focusing on the development of raw materials or low-value-added labor-intensive industries.

Compared with other cities, resource-based cities show a high dependence on natural resources, a certain cycle in the urban development process, loose urban spatial structure, relatively simple industrial structure, presenting relatively homogeneity and initial characteristics (9). In particular, the development of industries based on non-renewable resources is not sustainable, and resource-based cities that over-rely on these industries will lose their competitiveness as well as their attractiveness to capital and talents (21). The long-term development of a city largely depends on the use of resources (11), thus the sustainable development of a resource-based city requires an overall consideration of economic level, industrial structure, energy endowment, ecological efficiency, environmental protection and other aspects.

Scholars’ research on industrial structure upgrading and ecological environment mainly focuses on the following aspects: (1) The impact of environmental regulation on industrial structure upgrading (22–24). Numerous scholarly studies have found that environmental regulations can drive the upgrading of industrial structure, with the effect of innovation compensation outweighing that of compliance costs, and economic measures tend to be more effective than non-economic measures in achieving this objective (12, 18, 19, 25). In addition, some scholars have studied the impact of green finance, digital economy, technological innovation, low-carbon city pilot, innovative city pilot, sustainable development and other policies on industrial structure upgrading (9, 20, 26–29). (2) The impact of industrial structure upgrading on energy efficiency and carbon emissions (14, 17, 30, 31). Some scholars have shown that regional industrial structure differences have a significant impact on carbon emissions (32), especially in resource-based areas (33). The optimization and transformation of industrial structure is an important way to achieve low-carbon economic development in China (34). Green technological innovation can transform the energy-intensive industrial structure and adjust the proportions of the three major industries, thereby achieving energy conservation, emission reduction, and improved ecological efficiency (4, 5, 12, 34–36). (3) The interrelationship between industrial structure and natural resources and energy endowments (21, 30, 37). According to the research of some scholars, it has been proved that energy endowment will affect the evolution of regional industrial structure, which shows that regions with abundant natural resources and advantages of energy endowment are prone to ignore technological progress (38), resulting in resource dependence, and eventually forming a single energy-intensive industrial structure (39), and attracting a large amount of capital and human resources into energy-oriented (40). However, energy endowment is also one of the important factors that lead to the overexploitation of natural resources and the increase of carbon emissions (41).

Based on the research of previous literature on the research models and methods related to this topic, it is found that more traditional evaluation methods include grey relational degree analysis (42–44), fuzzy comprehensive evaluation method (45–48) and principal component analysis (49–51), among which the first two methods were mainly used to evaluate an individual system and cannot reflect the coordination relationship between multiple systems. While to evaluate the coordination relationship between two or more dynamically changing systems, the coupling coordination degree model was commonly used (26, 52–55).

Through the study of the existing literature, it is found that there are few quantitative studies on resource-based cities, especially for the resource-based cities in the three provinces of Northeast China, and the studies on the coupling and coordination relationship between industrial structure upgrading and ecological environment are also insufficient. Taking 19 resource-based cities in Northeast China as examples, this study supplements the existing researches in the following aspects: (1) Industrial structure upgrading was decomposed into industrial structure rationalization (RIS) and industrial structure advancement (AIS), and RIS was measured by improved Thiel index. (2) The EE evaluation index system was constructed from the three dimensions of ecological environment status, ecological environment pressure and ecological environment protection, and factors such as representativeness and national policy orientation were comprehensively considered in the selection of indicators. (3) The measures of RIS and AIS were coupled with the ecological environment subsystem respectively, and the results are more conducive to analyzing the internal mechanism of the interaction between industrial structure upgrading and EE in resource-based cities. (4) The research objects are 19 resource-based cities in Northeast China. Based on the coupling coordination degree model, a more matched coupling coordination path between the two subsystems was studied to provide policy suggestions for sustainable urban development, which is of great significance for the revitalization strategy of the old industrial base in Northeast China. The research conclusions can also be extended to other resource-based cities.

## 3 Study area and data sources

### 3.1 Study area

**Figure 2:**
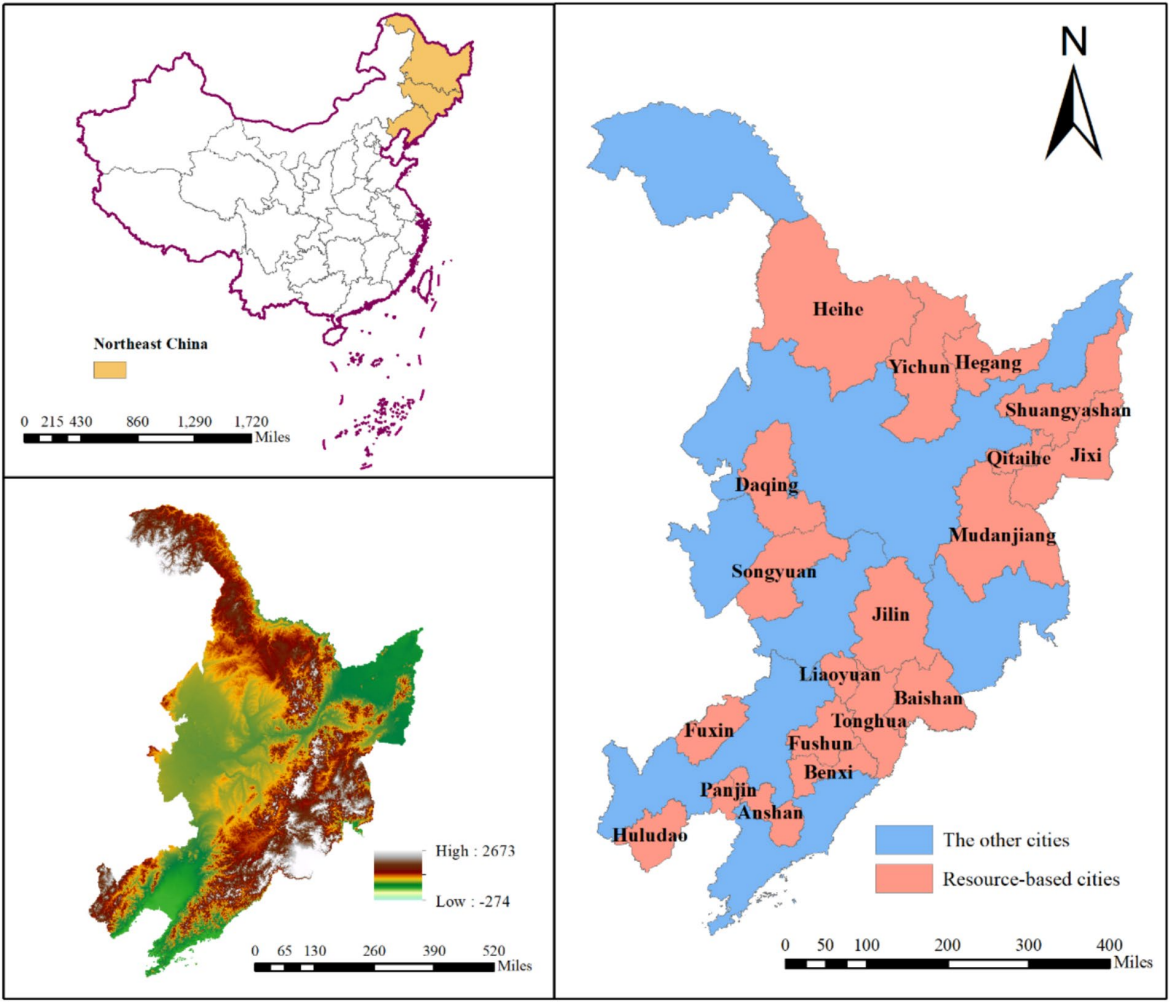
Regional location of resource-based cities in Northeast China.

Northeast China refers to the three northeastern provinces, including Liaoning, Jilin and Heilongjiang, with a total area of about 787,300 square kilometers and rich in natural resources. However, the depletion of natural resources caused by over-exploitation has become an important factor restricting the sustainable development of cities in Northeast China. *The National Plan for Sustainable Development of Resource-Based Cities (2013–2020)* promulgated by The State Council in 2013 listed in detail the list of resource-based cities across the country and made a comprehensive classification. In Northeast China, 19 prefecture-level cities were included in the list, including Fuxin, Fushun, Benxi, Anshan, Panjin and Huludao in Liaoning, Songyuan, Jilin, Liaoyuan, Tonghua and Baishan in Jilin, and Heihe, Daqing, Yichun, Hegang, Shuangyashan, Qitaihe, Jixi and Mudanjiang in Heilongjiange (Figure 2). Among them, only Songyuan belongs to growing resource-based city, accounting for only 5.26%, 6 cities belong to the mature resource-based city, accounting for 31.58%, as many as 8 cities in declining resource-based city, accounting for 42.11%, and 4 cities in the regenerative resource-based city, accounting for 21.05%. The study area is shown in Figure 2.

### 3.2 Data sources

The detailed sources of the data used in this study are shown in Table 1. The data of administrative divisions are from the official website of the National Basic Geographic Information Center. Some of the missing data were supplemented using the National Economic and Social Development Statistical Bulletins of each city from 2011 to 2022. For indicators with missing data in the first and last years, the linear trend method based on adjacent data was applied. Missing values for intermediate years were filled using linear interpolation, and for data that could not be calculated, the time series mean was employed to complete the dataset.

**Table 1:** Detailed sources of data.

Data sources	Indicators	Unit
Liaoning Statistical Yearbook (2011–2022); Jilin Statistical Yearbook (2011–2022); Heilongjiang Statistical Yearbook (2011–2022)	Output of primary, secondary and tertiary industries	Yuan
	Employment in primary, secondary and tertiary industries	Person
China Urban Statistical Yearbook (2011–2022)	Per capita water resources	m^3^/person
	Per capita public green area	m^3^/person
	Green coverage rate of built-up area	%
	Sewage treatment rate	%
	Harmless treatment rate of household garbage	%
China Urban Construction Statistical Yearbook (2011–2022)	Comprehensive utilization rate of industrial solid waste	%
	Per capita industrial wastewater discharge	Ton/person
	Per capita production of industrial solid waste	Ton/person
	Per capita industrial emissions	Ton/person

## 4 Index system construction and methods

### 4.1 The construction of indicator system

#### 4.1.1 Industrial structure upgrading index system

##### 4.1.1.1 Industrial structure rationalization

The industrial structure rationalization refers to the proportional relationship and the degree of coordination among various industries, which reflects the reasonable allocation or effective utilization of production factors among enterprises. Some scholars use the structural deviation degree to construct the RIS index, but this method regards the economic conditions of various industries as the same and ignores the relative importance of different industries, which has certain limitations. Theil index can overcome this shortcoming, avoid the calculation of absolute values (56), and retain the theoretical basis and economic implications of structural deviation (20). Thiel index was used initially to measure the degree of income inequality between individuals. With the deepening of the research, it is also gradually used to measure the disparity between regions in terms of income, economic development and green development. Similarly, in this paper, the larger the value of Thiel index, the more irrational the industrial structure. The improved Thiel Index was adopted as the index of industrial structure rationalization, and the calculation formula is shown in Equation 1:


(1) $$RIS=\mathrm{TL}=\overset{3}{\underset{i=1}{\sum }}\left(\frac{Y_{i}}{Y}\right)\ln\left(\frac{Y_{i}}{L_{i}}/\frac{Y}{L}\right)$$

TL represents Theil index; Y represents the total output value; L represents the total number of employment, and i represents the industry ( i=1,2,3 ). When TL=0 , it indicates that the economy is in an equilibrium state, and the larger the TL value, the more the industrial structure deviates from the equilibrium state and the more unreasonable the industrial structure.

##### 4.1.1.2 Industrial structure advancement

The industrial structure advancement refers to the dynamic process of the development of industrial structure from low technology level to high technology level, which is manifested in the sequence of industrial added value from “one, two, three” to “three, two, one,” that is, the output value of the tertiary industry accounts for an increasing proportion of GDP, and the industrial structure develops in the direction of “service oriented” in the process of economic development. Some scholars also believe that the development of the tertiary industry reflects clean and green production to some extent (57, 58), and the industrial structure advancement can be measured by the proportion of the tertiary industry. Based on relevant studies (27, 59), this study used the output value ratio of the tertiary industry and the secondary industry to measure the industrial structure advancement. The calculation process is shown in Equation 2:


(2) $$AIS=\frac{Y_{3}}{Y_{2}}$$

$Y_{2}$ represents the output value of the secondary industry and $Y_{3}$ represents the output value of the tertiary industry. The larger the AIS value is, the larger the proportion of the tertiary industry to the secondary industry, the more service-oriented the industrial structure, the more advanced the industrial structure, and the greater the proportion of high-tech and high value-added industries.

#### 4.1.2 Ecological environment

Ecological environment refers to the quantity and quality of water resources, land resources, biological resources and climate resources that affect human survival and development, and is a composite ecosystem related to sustainable social and economic development. Therefore, to construct the ecological environment index system, it is necessary to fully consider the natural elements such as water, soil and gas, and also consider the interaction between the ecological environment and social and economic development. Drawing on the method of Xiao et al. (55), this study selected 9 secondary indicators from three aspects of ecological environment status, ecological environment pressure and ecological environment protection to construct the indicator system of ecological environment (Table 2). The ecological environment status is a positive index, which can reflect the resource endowment and ecological environment status of a region to some extent. The ecological environment pressure is a negative index, which mainly considers the degree of pollution and damage of industrial development to the environment to measure the bearing pressure of the ecological environment. Ecological environment protection is also a positive indicator, which can reflect the intensity and effect of environmental regulation on ecological environment in a region.

**Table 2:** Ecological environment evaluation index system.

Primary indicator	Indicators	Unit	Attribute	Weight
Ecological environment level	Per capita water resources	m^3^/person	+	0.2353
	Per capita public green area	m^3^/person		0.1973
	Green coverage rate of built-up area	%		0.0826
Ecological environment pressure	Per capita industrial wastewater discharge	Ton/person	−	0.0778
	Per capita production of industrial solid waste	Ton/person		0.0469
	Per capita industrial emissions	Ton/person		0.0851
Ecological environment protection	Sewage treatment rate	%	+	0.0818
	Harmless treatment rate of household garbage	%		0.0609
	Comprehensive utilization rate of industrial solid waste	%		0.1323

The weight coefficients in the last column of this table are calculated from the entropy-Critic combined weight model mentioned in the following article.

### 4.2 Entropy–CRITIC–TOPSIS model

Entropy method is an objective weighting method, which determines the weight according to the variability of an indicator. This approach not only highlights the significance of each indicator in the decision-making process and preserves its original information but also captures changes in indicator weights over time. As a result, it reduces subjective bias and enhances the scientific rigor of evaluations (60). Indicators with greater variability convey more information and are therefore assigned higher weights.

The CRITIC weighting method assigns weights by introducing contrast intensity and conflict to quantify the informational content of each evaluation indicator (61). Contrast intensity, based on the concept of mean square error, reflects the variation among indicators, while conflict is measured using the correlation coefficient, with higher correlation coefficients indicating lower levels of conflict. This method is particularly well-suited for datasets with certain correlation between the indicators, aligning effectively with the structure of the indicator system developed in this study.

Both methods have inherent limitations when applied independently. However, combining the entropy weight method with the CRITIC method enables a more balanced consideration of factor weights and their interrelationships. The entropy weight method effectively calculates factor weights, while the CRITIC method provides a comprehensive analysis to determine the relationships between factors. This integrated approach offers a more accurate foundation for decision-making in the analytical process. Therefore, in this study, a combined weighting model integrating the entropy method and the CRITIC method will be employed to assign weights to ecological environment assessment indicators.

TOPSIS method is a scientific evaluation method first proposed by Hwang and Yoon (28, 62) and Zhao et al. (28). It selects the best scheme by ranking the finite evaluation objects according to their proximity to the idealized target. The method employed in this study integrates the strengths of the entropy method, the CRITIC method, and the TOPSIS method to ensure a more scientific and reasonable evaluation (63). Specifically, the combined weights of each indicator are first calculated using the entropy and CRITIC methods to construct a standardized weighting matrix, which is then incorporated into the TOPSIS model for a comprehensive evaluation of the system. Entropy-CRITIC-TOPSIS method’s objectivity mitigates bias stemming from subjective assignment and is suitable for the scientific evaluation of the system.

#### 4.2.1 Entropy-critic combination weight model

(1) Standardization of raw data:

Assuming that there are m evaluation objects and n evaluation indicators in the system, the initial evaluation matrix is $X={\left(x_{ij}\right)}_{m\times n}$ ( i=1,2,⋯,m and j=1,2,⋯,n ), and $x_{ij}$ represents the value of the i index of the j evaluation object. The extreme value treatment method adopted by the predecessors was used to eliminate its dimension.

If $x_{ij}$ is a positive indicator, the calculation process is shown in Equation 3:


(3) $$x_{ij}^{'}=\frac{x_{ij}-x_{\text{min}}}{x_{\text{max}}-x_{\text{min}}}+0.0001$$

If $x_{ij}$ is a negative indicator, the calculation process is shown in Equation 4:


(4) $$x_{ij}^{'}=\frac{x_{\text{max}}-x_{ij}}{x_{\text{max}}-x_{\text{min}}}+0.0001$$

(2) According to the entropy value method to calculate index weight:

a. Calculate the proportion of the index system (Equation 5):


(5) $$p_{ij}=\frac{x_{ij}^{'}}{\sum_{i=1}^{m}x_{ij}^{'}}$$

b. Calculate the entropy of each index (Equation 6):


(6) $$e_{j}=-\frac{1}{\ln m}\overset{m}{\underset{i=1}{\sum }}p_{ij}\ln p_{ij}$$

c. Calculate index weights (Equation 7):


(7) $$w_{j}^{'}=\frac{1-e_{j}}{\sum_{j=1}^{n}\left(1-e_{j}\right)}$$

(3) According to the CRITIC method to calculate index weight:

a. Calculate the correlation coefficient matrix among indicators (Equation 8):


(8) $$r_{ij}=\frac{\sum_{i=1}^{n}\left(x_{i}-\bar{x}\right)\left(y_{i}-\bar{y}\right)}{\sqrt{\sum_{i=1}^{n}{\left(x_{i}-\bar{x}\right)}^{2}\sum_{i=1}^{n}{\left(y_{i}-\bar{y}\right)}^{2}}}$$

The correlation coefficient matrix between indexes is $R={\left(r_{ij}\right)}_{m\times n}$ .

b. Calculate the amount of information for the indicator (Equation 9):


(9) $$c_{j}=\sigma_{j}\overset{n}{\underset{i=1}{\sum }}\left(1-r_{ij}\right)$$

$\sigma_{j}$ is the mean square deviation of the j indicator, reflecting the difference among indicators; $\overset{n}{\underset{i=1}{\sum }}\left(1-r_{ij}\right)$ reflects the conflict between the indicators; $c_{j}$ represents the amount of information contained in the indicator, and the larger the value, the larger the amount of information contained.

c. Calculate index weights (Equation 10):


(10) $$w_{j}^{''}=\frac{c_{j}}{\sum_{j=1}^{n}c_{j}}$$

(4) Calculate the combined weights of indicators (Equation 11):


(11) $$w_{j}=\beta w_{j}^{'}+\left(1-\beta \right)w_{j}^{''}$$

This study holds that CRITIC method is more objective and comprehensive, so it is given a higher weight. The ratio of entropy weight method and CRITIC method in the combined weight is 4: 6.

#### 4.2.2 TOPSIS model

(1) Construct a standardized weighting matrix (Equation 12):


(12) $$V={\left(V_{ij}\right)}_{m\times n}={\left(w_{j}x_{ij}^{'}\right)}_{m\times n}$$

(2) Define positive ideal solutions and negative ideal solutions (Equations 13, 14):


(13) $$V^{+}=\left(\begin{array}{l} \max\left\{v_{11},v_{21},\cdots ,v_{n1}\right\},\max\left\{v_{12},v_{22},\cdots ,v_{n2}\right\},\cdots ,\\ \max\left\{v_{1m},v_{2m},\cdots ,v_{\mathrm{nm}}\right\} \end{array}\right)$$

(14) $$V^{-}=\left(\begin{array}{l} \min\left\{v_{11},v_{21},\cdots ,v_{n1}\right\},\min\left\{v_{12},v_{22},\cdots ,v_{n2}\right\},\\ \cdots ,\min\left\{v_{1m},v_{2m},\cdots ,v_{\mathrm{nm}}\right\} \end{array}\right)$$

(3) Calculate the Euclidean distance between the i -th ( i=1,2,⋯m ) evaluation object and the positive and negative ideal solutions (Equations 15, 16):


(15) $$D_{i}^{+}=\sqrt{\overset{n}{\underset{j=1}{\sum }}{\left(v_{ij}-v_{j}^{+}\right)}^{2}}$$

(16) $$D_{i}^{-}=\sqrt{\overset{n}{\underset{j=1}{\sum }}{\left(v_{ij}-v_{j}^{-}\right)}^{2}}$$

(4) Calculate the relative proximity score of the i ( i=1,2,⋯m ) evaluation object (Equation 17):


(17) $$S_{i}=\frac{D_{i}^{-}}{D_{i}^{+}+D_{i}^{-}}0\leq S_{i}\leq 1$$

The larger the $S_{i}$ value, the smaller $D_{i}^{+}$ is, which indicates that the evaluation object is closer to the positive ideal solution, that is, closer to the optimal level, and vice versa.

### 4.3 Coupling coordination degree model

The CCD model is a valuable analytical tool for evaluating the degree of coordinated development among various entities. It enables a systematic assessment of the relationships, synergies, and coordination levels across different subsystems within a regional economic system. By capturing the dynamic interactions between these subsystems, the CCD model provides insights into the balance and interdependence necessary for sustainable and integrated development.

The CCD model was constructed to study the interaction and coordination between industrial structure upgrading and ecological environment of these 19 resource-based cities. By referring to the concept and model of capacity coupling in physics, the coupling degree function of interaction between two or more systems can be generalized (Equation 18):


(18) $$C_{n}=\frac{\sqrt[{n}]{U_{1}\times U_{2}\times \cdots \times U_{n}}}{U_{1}+U_{2}+\cdots +U_{n}}$$

$U_{i}$ represents the development level of each subsystem and $i\in \left(1,2,\cdots ,n\right)$ .

The coupling degree function of the two systems is (Equation 19):


(19) $$C=\frac{\sqrt{U_{1}\times U_{2}}}{U_{1}+U_{2}}$$

In order to avoid the situation that the development level of the two systems is low, but the coupling value is high, a coupling coordination degree model was constructed (Equations 20, 21):


(20) $$T=\alpha U_{1}+\beta U_{2}\left(\alpha ,\beta \in \left(0,1\right),\alpha +\beta =1\right)$$

(21) $$CCD=\sqrt{C\times T}$$

T represents the comprehensive development level of the two systems $U_{1}$ and $U_{2}$ ; α and β respectively represent the weight coefficient of subsystem $U_{1}$ and subsystem $U_{2}$ . In this study, it is considered that the industrial structure upgrading is as important as the ecological environment, so take α=β=0.5 . CCD represents the coupling coordination degree between the two systems, and the value range is [0, 1]. The greater the CCD value, the higher the degree of coordination between the systems; on the contrary, the more inconsistent. Based on the classification of Chen et al. (53), Li et al. (26), and Ariken et al. (52), this study divided the classification into 5 levels, as shown in Table 3.

**Table 3:** Classification of coupling coordination degree.

Criteria	Classification	Stage
(0.8, 1)	Extreme coordination	Coordination development
(0.6, 0.8)	Moderate coordination	Transformation development
(0.4, 0.6)	Basically coordination	
(0.2, 0.4)	Moderate imbalanced	Uncoordinated development
(0, 0.2)	Serious imbalanced	

## 5 Results

### 5.1 Results analysis of the industrial structure subsystem

#### 5.1.1 Industrial structure rationalization

The RIS level of 19 resource-based cities in Northeast China showed a fluctuating increase from 2010 to 2021, with the average value rising from 0.102 in 2010 to 0.293 in 2020, and slightly decreasing to 0.267 in 2021, indicating that the unreasonable industrial structure is aggravating year by year, and there is unbalanced development among different cities. The main reason for the RIS level may be that the Northeast China have been dominated by traditional economic development models for many years, especially in these resource-based cities, where the industrial structure is relatively single and dominated by resource-dependent industries such as energy, mining, and heavy industries. Such an industrial structure will lead to excessive exploitation and even waste of resources, which is not in line with the Chinese sustainable development strategy. Promoting the adjustment and rationalization of the industrial structure of resource-based cities in Northeastern China still faces significant challenges.

**Figure 3:**
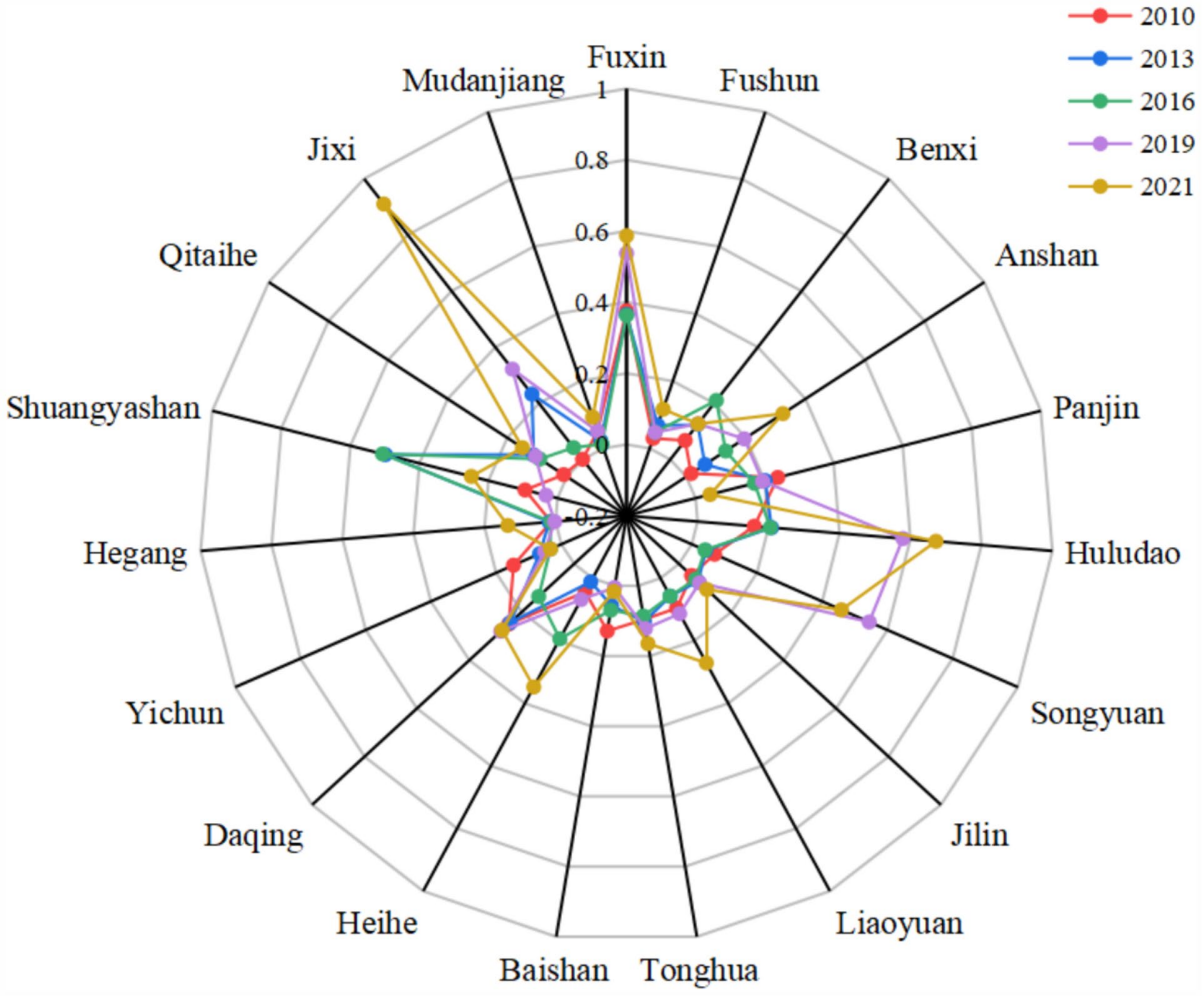
Industrial structure rationalization level.

There are great differences in RIS among 19 resource-based cities (Figure 3). The average RIS index of each city ranges from 0.026 (Hegang) to 0.461 (Fuxin), and 7 cities (Fuxin, Panjin, Hulu, Songyuan, Daqing, Shuangyashan and Jixi) were higher than the average RIS of 0.155, accounting for 36.8%. These cities face heightened pressure for industrial restructuring and transformation, with Fuxin experiencing the most severe challenges. As the city with the highest RIS index, Fuxin grapples with issues such as a narrow industrial structure, fragmented industrial chains, and a lack of distinctive industrial features. In response, local authorities have actively sought solutions, implementing strategies in recent years to transition from a “coal-dominated” economy to a diversified and synergistic cluster development model.

Hegang exhibits the highest RIS level among the cities, largely attributed to its effective policy leadership. In its “14th Five-Year Plan,” Hegang emphasized the integrated development of industries, focusing on six key directions: coal, graphite, modern agriculture, green industries, the strategic new economy, and the cultivation of emerging industrial forms. The city also aims to establish itself as a “demonstration city for industrial transformation.” These targeted policies have significantly accelerated Hegang’s pace of industrial transformation.

#### 5.1.2 Industrial structure advancement

**Figure 4:**
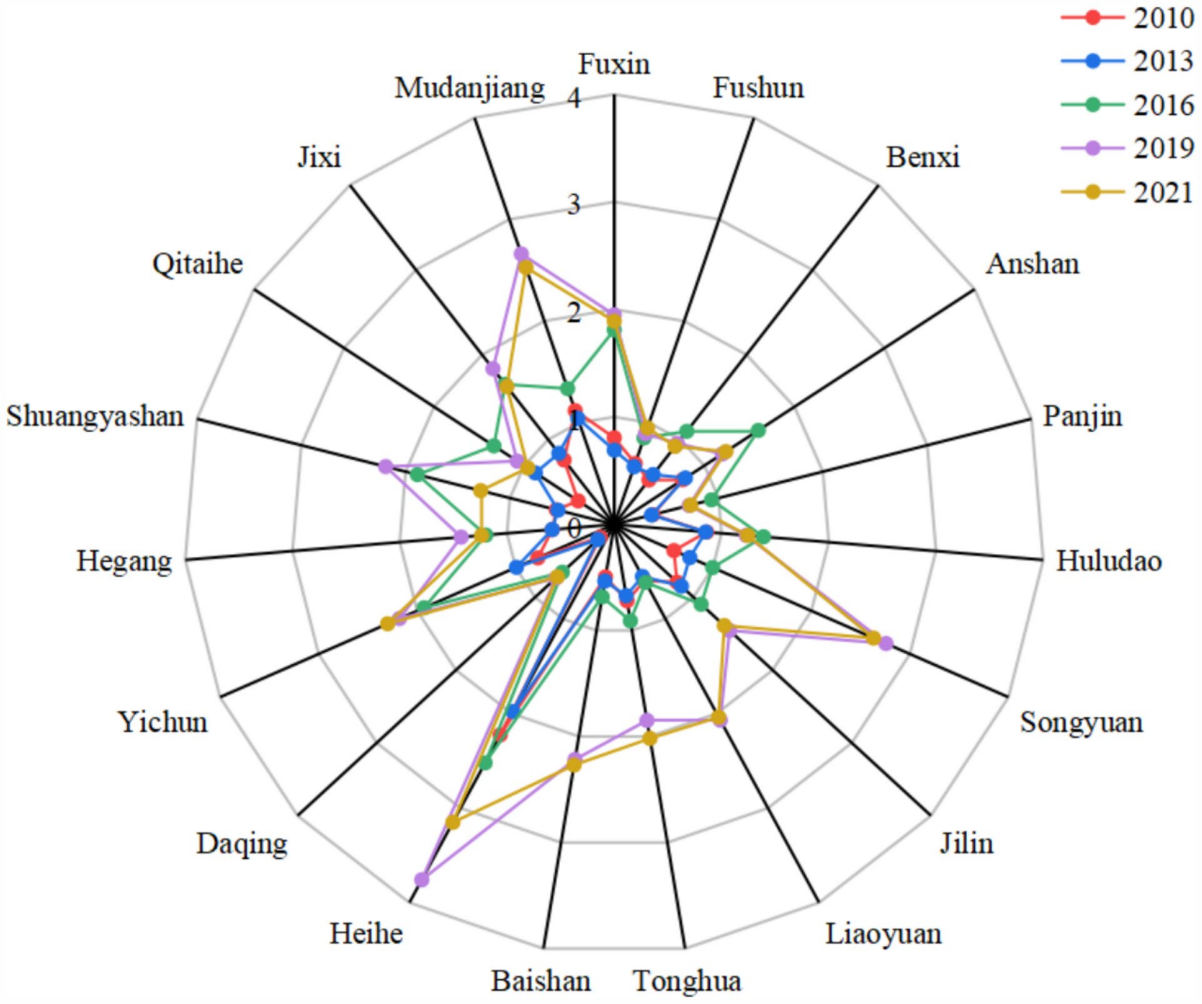
Industrial structure advancement level.

From 2010 to 2019, the industrial structure of 19 resource-based cities in Northeast China showed a steady upward trend, rising from 0.722 in 2010 to 1.745 in 2019. From 2010 to 2018, the average annual growth rate was only 8.62%, but in 2018–2019, the growth rate reached 24.73%. The industrial structure advancement level is shown in Figure 4. The possible reason is that the national and local governments have issued a series of policies to encourage and support resource-based cities to adjust their industrial structure and develop emerging industries and modern service industries. The rapid development of modern service industries such as finance, logistics, tourism and culture in some cities has injected new vitality into the growth of the tertiary industry. Coupled with the fact that resource-based cities are facing the gradual depletion of resources and increasing environmental pressure under the strategic background of national sustainable development, some high-polluting and high-energy-consuming enterprises are forced to shut down or transform, further weakening the strength of the secondary industry. The double reasons make the ratio of the added value of the tertiary industry and the secondary industry increase further. Due to a certain lag effect of the policy, this increase was highlighted in 2018–2019. However, AIS began to decline in 2020 and increase in 2021, which may be due to the severe impact of the COVID-19 pandemic on service industries such as catering, cultural tourism, and transportation in these cities.

The average AIS value of all 19 cities was 1.173, only 4 cities (Fuxin, Songyuan, Heihe and Yichun) were higher than the average AIS value, and the city with the highest score was Heihe in Heilongjiang Province (2.553). The city with the lowest score was Daqing in Heilongjiang, which was only 0.490. For a long time, Daqing’s economic development mainly relies on the oil industry, and the secondary industry dominates the economy. Although the local government and enterprises have made efforts to promote industrial diversification in recent years, the tertiary industry growth rate is higher than the national average, but compared with the secondary industry, the proportion of the tertiary industry is still low, and it faces technical and market challenges.

Differences in city policies are a key factor influencing variations in industrial structure advancement among cities. Heihe has closely aligned its development with Heilongjiang Province’s “4,567” modern industrial system, leveraging its industrial base and a well-defined strategic layout. Efforts have been focused on building the “three major industrial clusters,” “three advantageous industries,” and “four new economy sectors.” A series of key industrial projects have been initiated and accelerated, contributing to continuous improvements in the quality of economic development. In contrast, Daqing’s industrial focus remains centered on the oil industry. At the 2024 year-end work conference, the city reiterated its commitment to seeking breakthroughs in the integration and development of central and local governments while providing comprehensive support for shale oil exploration and development.

### 5.2 Results analysis of the ecological environment subsystem

**Figure 5:**
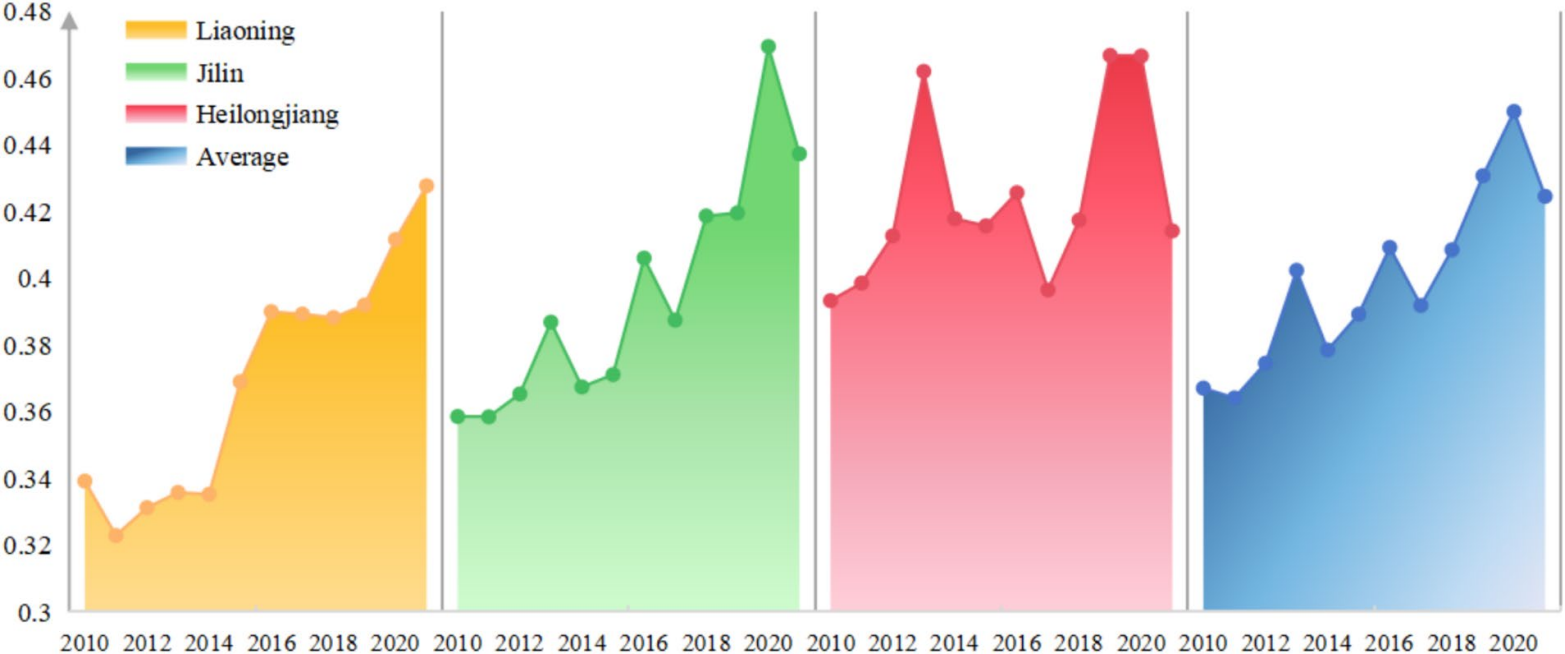
Mean value of comprehensive ecosystem evaluation score (2010–2021).

The comprehensive level of ecological environment in 19 cities from 2010 to 2021 showed a fluctuating and slow rise (Figure 5), with the average value increasing from 0.3670 in 2010 to 0.4245 in 2021, and the average annual growth rate was 1.33%, with slight declines in some years. For instance, there was a decline of 0.79% from the previous year in 2011, 5.92% in 2014, 4.25% in 2017, and 5.65% in 2021. The highest growth rate was 7.42% in 2012–2013, which was much higher than the average growth rate, which may be attributed to the concept of building a “beautiful China” put forward at the 18th National Congress of the Communist Party of China, emphasizing that the construction of ecological civilization should be placed in a prominent position and integrated into all aspects and the whole process of economic, political, cultural and social construction.

Relevant policies have been quickly implemented in various cities, so that all aspects of ecological environment have been significantly improved in these resource-based cities. For example, since 2020, Benxi City has embraced the vision of becoming an “ecological city” by launching three major environmental campaigns focused on preserving green mountains, achieving blue skies, and safeguarding clear waters. These efforts aim to establish Benxi as the “Green Steel Capital” and the “Northern Pharmaceutical Valley,” driving sustainable improvements in environmental quality. Similarly, Yichun has explored innovative pathways to realize the value of ecological products. The city has consistently increased investments, with a total of 98.041 billion yuan allocated to support the robust development of projects such as ecotourism, forest-based food industries, and northern medicinal plant cultivation.

**Figure 6:**
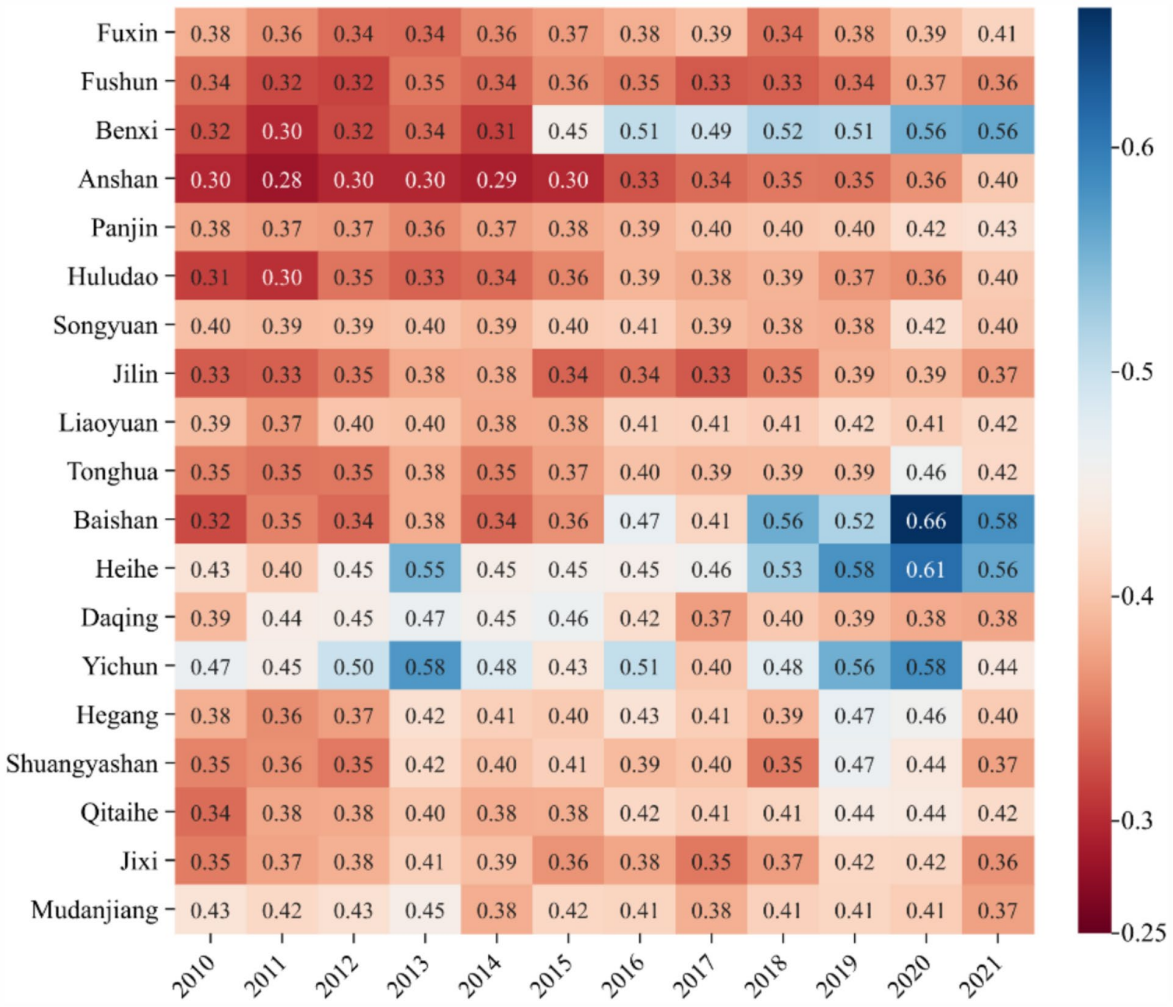
The heatmap of EE evaluation level.

Among individual cities (Figure 6), Heihe in Heilongjiang ranks highest with a score of 0.494, while Anshan in Liaoning ranks lowest at 0.324. There was little difference between the cities. However, growth rates reveal significant disparities. Baishan in Jilin exhibited the largest increase, rising by 81.92% from 0.3202 in 2010 to 0.5825 in 2021. In recent years, Baishan has actively pursued a development path emphasizing ecological priorities, resource conservation, intensification, and green, low-carbon growth. The city has made significant strides in building a resilient and sustainable ecological urban model, achieving notable results. In 2017, Baishan was recognized as a typical for green transformation in the National Development and Reform Commission’s series of reports on “The Transformation and Development of Resource-Based Cities.” The report highly commended Baishan’s efforts in fostering industrial clusters, promoting ecological livability, and advancing reform and opening-up initiatives. In contrast, Mudanjiang of Heilongjiang, experienced a slight deterioration of its ecological environment in the past 12 years, with its score dropping 13.15% from 0.4301 in 2010 to 0.3735 in 2021, which may be due to the phenomenon of “emphasizing development over environmental protection” in the process of promoting economic development, as well as inadequate environmental management.

### 5.3 Results analysis of coupling coordination degree

#### 5.3.1 Results analysis of coupling coordination degree of RIS and EE

**Figure 7:**
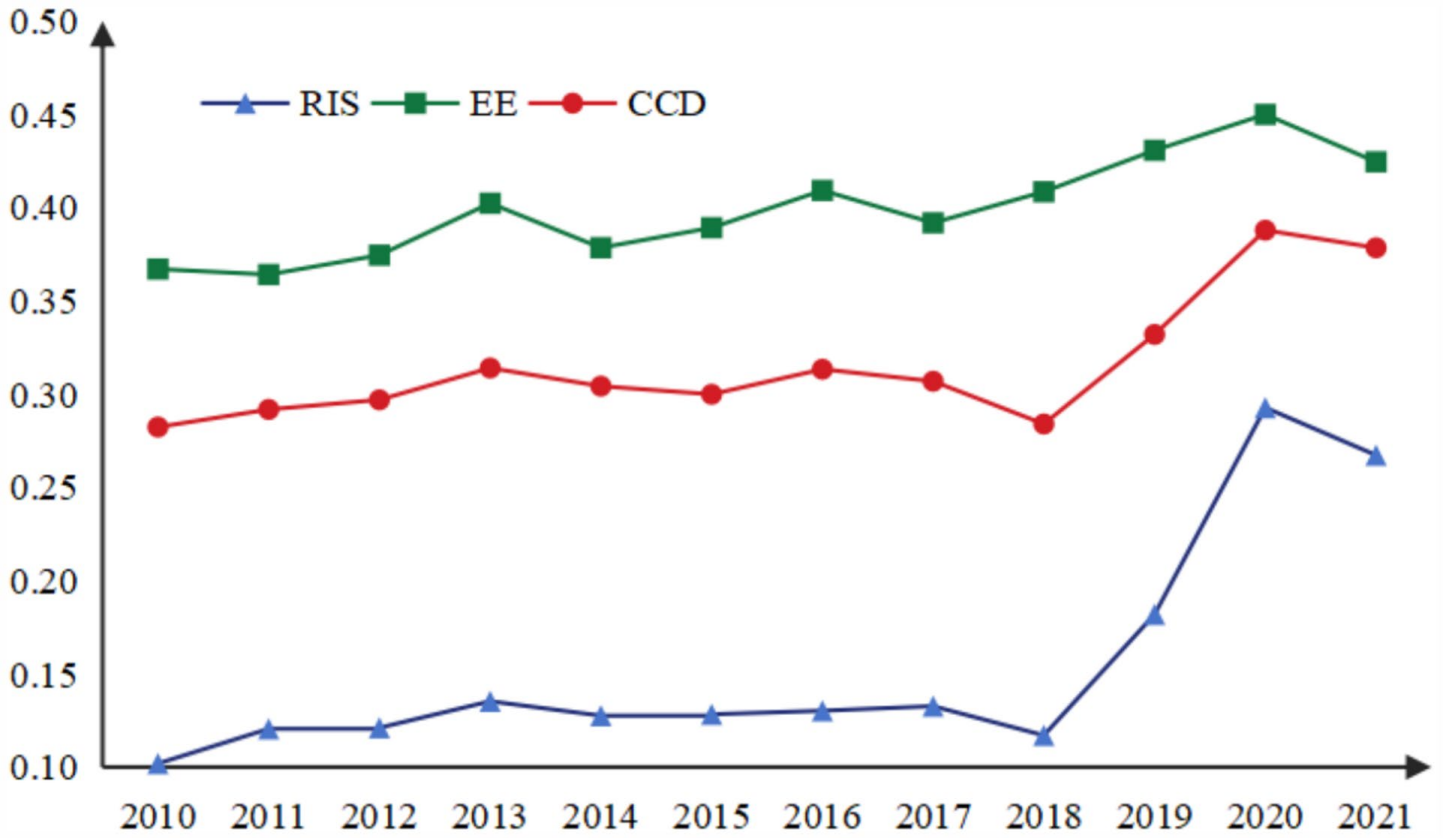
The CCD of RIS and EE.

**Figure 8:**
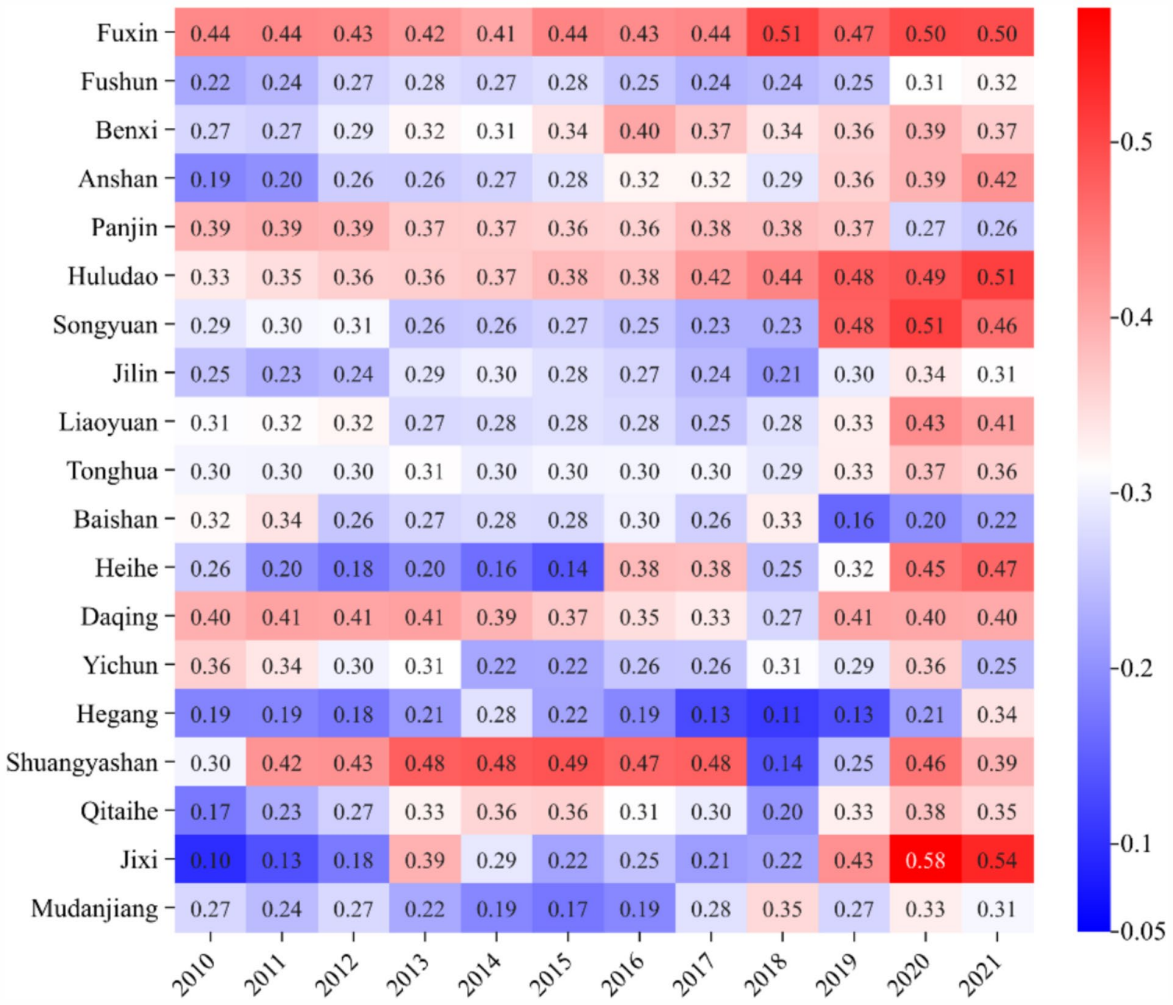
The heatmap of CCD of RIS and EE.

**Figure 9:**
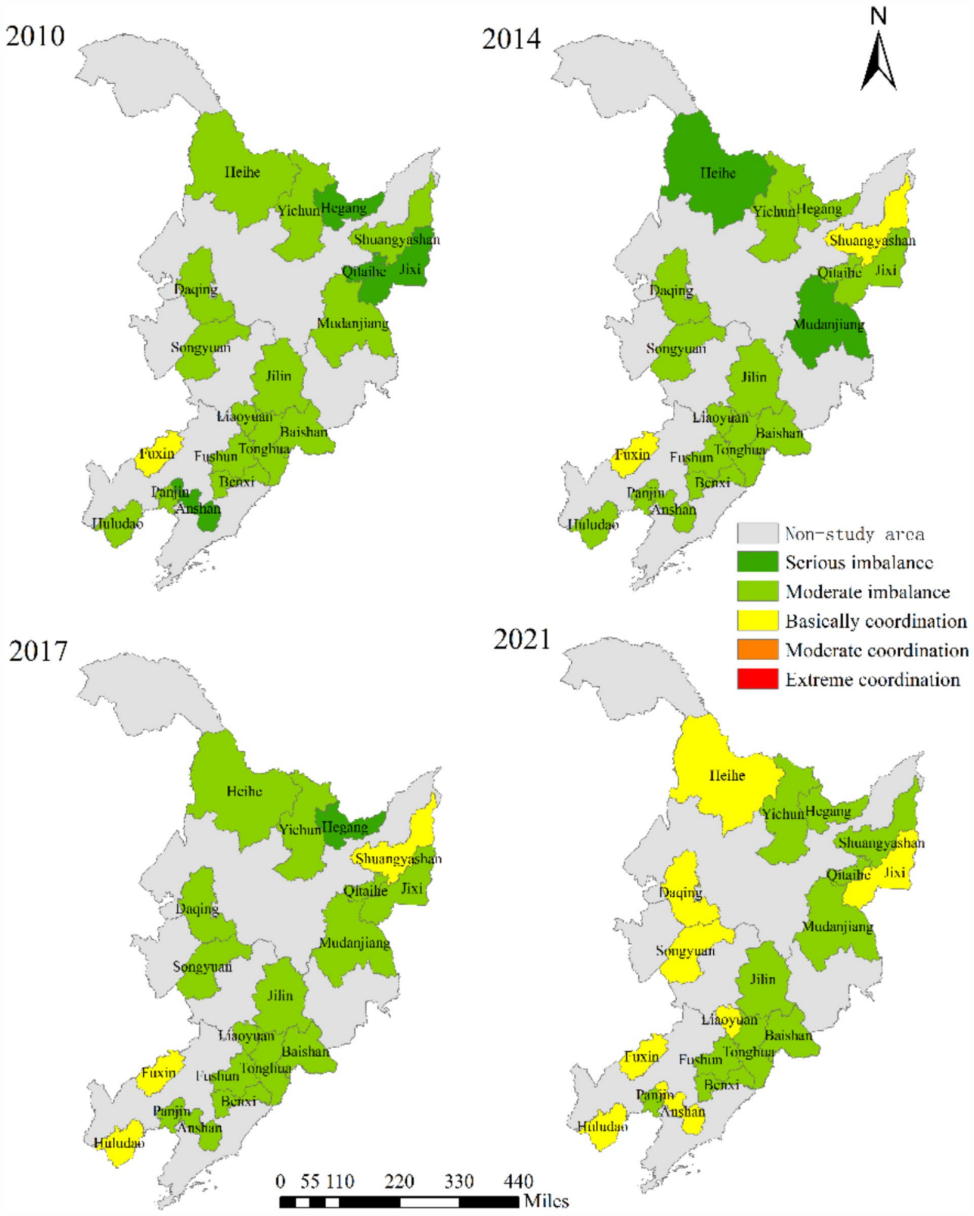
The spatial–temporal evolution of CCD of RIS and EE of 19 resource-based cities.

The coupling coordination degree of RIS and EE in 19 resource-based cities showed an overall increasing trend (Figures 7, 8). From 2010 to 2018, the change range of CCD was small, while from 2018 to 2020, the annual average growth rate reached 16.87%, and then showed a downward trend in 2021. From the spatial–temporal evolution perspective (Figure 9), the coordinated development trend of industrial structure rationalization and ecological environment system of these 19 resource-based cities in Northeast China was moderate imbalanced. Only two cities, Fuxin (0.4515) and Huludao (0.4057) in Liaoning, achieved basic coordination. In addition, Hegang in Heilongjiang was in a serious imbalanced state, with an average CCD of 0.1978 from 2010 to 2021, which was the lowest among all 19 cities. However, by examining the development trend of CCD, it will be found that Hegang had improved from serious imbalance to moderate imbalance since 2020, and the coupling coordination degree of RIS and EE had also increased year by year to 0.3416 in 2021, which may also be due to the awareness of the importance of the coordinated development of industrial structure and ecological environment. The proportion of CCD in the five categories of 19 cities was 0: 0: 2: 16: 1, and the proportion of industrial structure rationalization and ecological environment system development was not coordinated was as high as 89.47%, and the transformation and upgrading of industrial structure and ecological environment protection of resource-based cities are imminent.

The variations in CCD of RIS and EE are primarily driven by inter-city differences. Hegang and Fuxin provide illustrative examples. In Hegang, the severe imbalance between industrial structure rationalization and ecological environment development is largely due to a lack of synergy between its industrial and environmental policies. While Hegang demonstrates distinctive perspectives on industrial planning, its reliance on coal as the dominant industry has resulted in ongoing ecological degradation. In contrast, Fuxin exhibits a relatively balanced industrial structure rationalization and ecological environment coordination. However, this performance suggests that Fuxin faces challenges in both areas to some extent. According to the analysis presented in this study, Fuxin has achieved notable improvements in both industrial rationalization and ecological environment development over the past two years, signaling its transition toward a more coordinated development path.

#### 5.3.2 Results analysis of coupling coordination degree of AIS and EE

**Figure 10:**
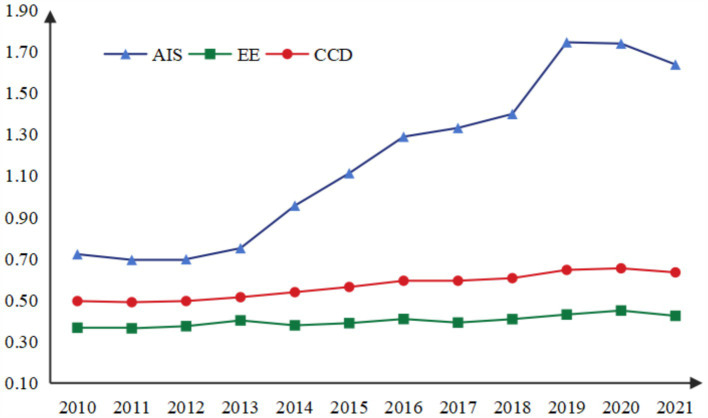
The CCD of AIS and EE.

**Figure 11:**
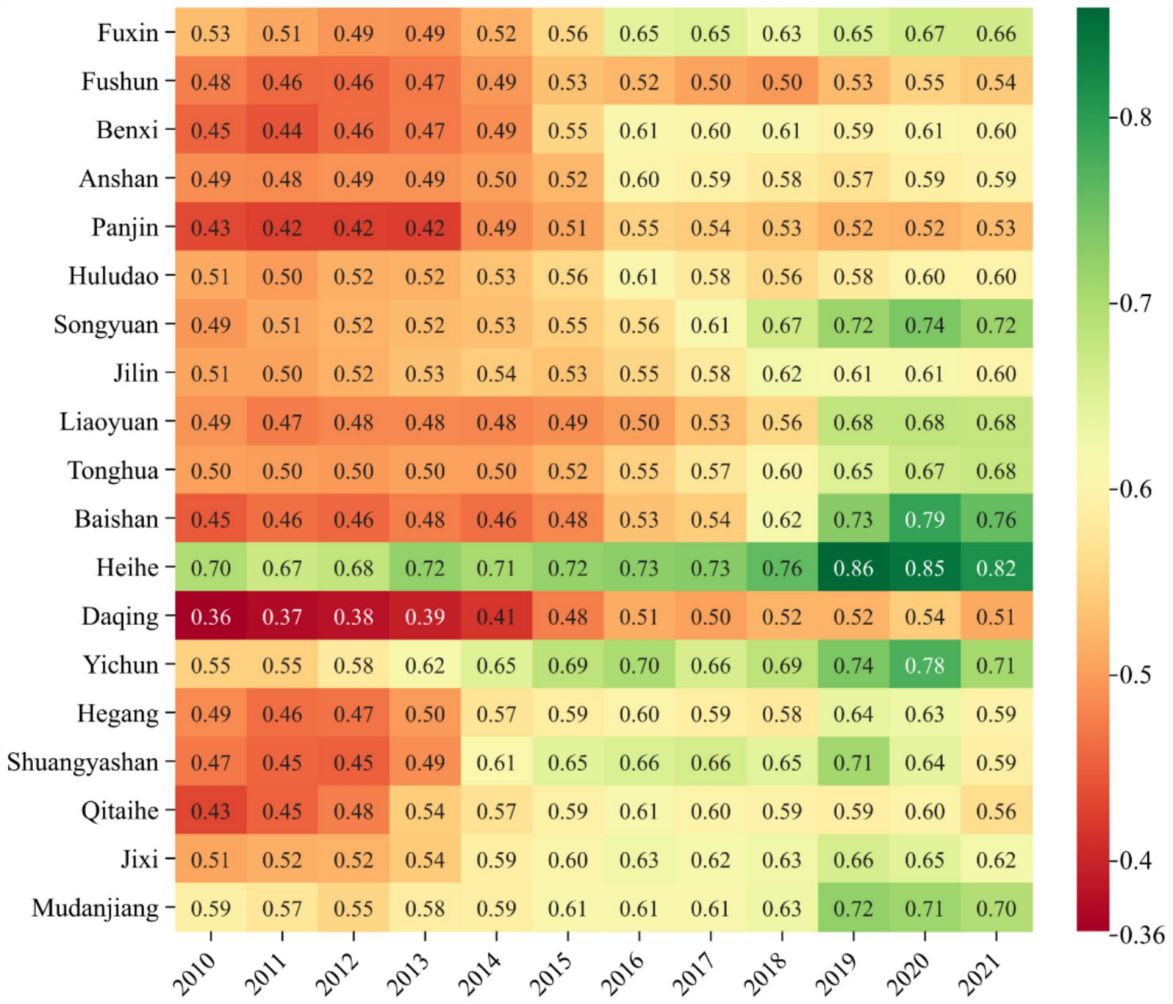
The heatmap of CCD of AIS and EE.

**Figure 12:**
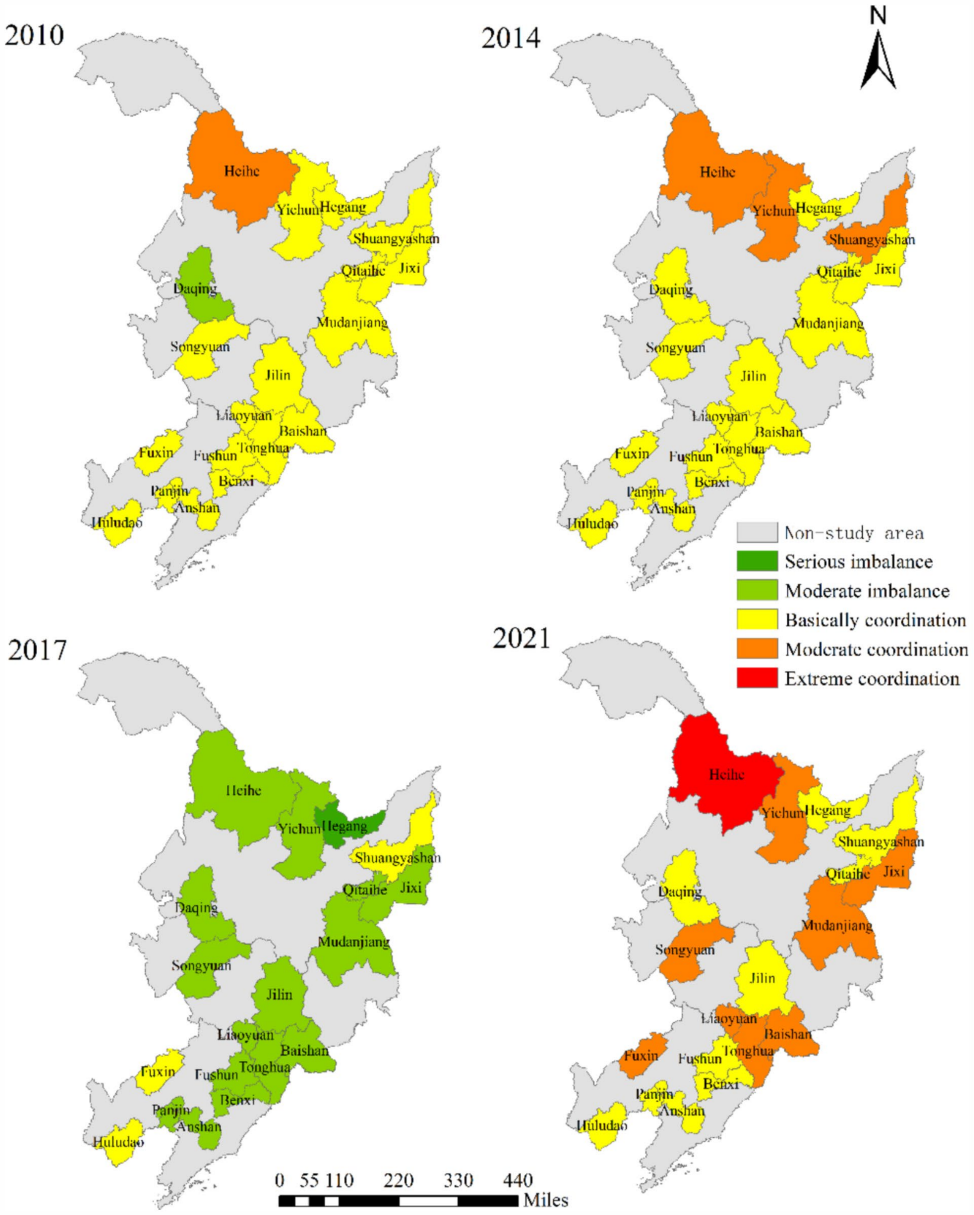
The spatial–temporal evolution of CCD of AIS and EE of 19 resource-based cities.

The coupling coordination degree of AIS and EE in 19 resource-based cities showed an overall increasing trend, but the increase was small (Figures 10, 11). Compared with the CCD of RIS and EE, the CCD of AIS and EE was relatively high. According to the spatial–temporal evolution (Figure 12), 19 resource-based cities were in basic coordination or above, and the CCD average of 3 cities (Heihe, Yichun and Mudanjiang) exceeded 0.6, reaching moderate coordination, accounting for 15.79%.

Heihe recorded the highest CCD value (0.746), and since 2019, its CCD of AIS and EE has consistently fallen within the extreme coordination range. In recent years, Heihe has successfully aligned with emerging economic development trends, demonstrated a willingness to innovate, and leveraged its unique resource endowments and industrial base to implement green and intelligent transformations in its industrial structure. The city has also undertaken targeted environmental protection initiatives, such as the “Green Shield” nature reserve inspections and the “three-line, one-order” ecological zoning control system, which strictly enforces ecological protection red lines. These measures have significantly enhanced the collaborative development of AIS and EE. In contrast, Daqing in Heilongjiang registered the lowest CCD value (0.459). Between 2010 and 2013, its CCD remained below 0.4, indicating a state of moderate imbalance. Since 2014, it had improved, but there were still fluctuations in some years. The month-on-month decline in 2021 was 5.99%, almost double the average decline (3.01%) for all cities in that year.

The fluctuation in AIS was more pronounced than that in EE. Despite these challenges, the overall CCD development trend was stable and positive. This reflects that over the past decade, the 19 resource-based cities in Northeast China have increasingly prioritized the growth of the tertiary sector while making efforts to protect the ecological environment during industrial transformation. These actions have contributed to healthier and more coordinated development of the AIS and EE systems.

#### 5.3.3 Results analysis of spatial relationship

##### 5.3.3.1 Result analysis of geo-detector

Numerous studies have proven that changes in the ecologic environment are often closely related to human factors (64–66). In order to explore the factors affecting the coupling degree of industrial upgrading and ecological environment, we leveraged relevant extant literature and select four variables as shown in Table 4. Subsequently, the Geo-detector was used to explore the influence degree and significance of each variable.

**Table 4:** Description of geo-detector variables.

Variables	Description
Economic capacity (X1)	GDP per capita
Capability of communication (X2)	Number of telephone communications
Density of population (X3)	Ratio of population to urban area
Infrastructure capacity (X4)	The number of books in the library

As shown in Table 5, in the single-factor detection, focusing on the coupling degree of RS and EE, it can be found that the selected four variables all pass the significance test at 5% level, which proves that economy, communication, population and infrastructure all have obvious effects on the coupling degree of RS and EE. Among them, population density has the strongest influence on the coupling degree, The possible explanation is that the increase of population density is often accompanied by the acceleration of urbanization, which will lead to drastic changes in industrial structure. In the detection of AIS and EE coupling degree, only economy and population pass the significance test, and population is still the factor with the highest degree of influence.

**Table 5:** Single factor detection results.

	Q (RS - EE)	P (RS - EE)	Q (AIS - EE)	P (AIS - EE)
Economic capacity	0.071	0.030**	0.189	0***
Capability of communication	0.112	0.006***	0.035	0.903
Density of population	0.227	0.000***	0.373	0***
Infrastructure capacity	0.059	0.021**	0.014	0.751

**Figure 13:**
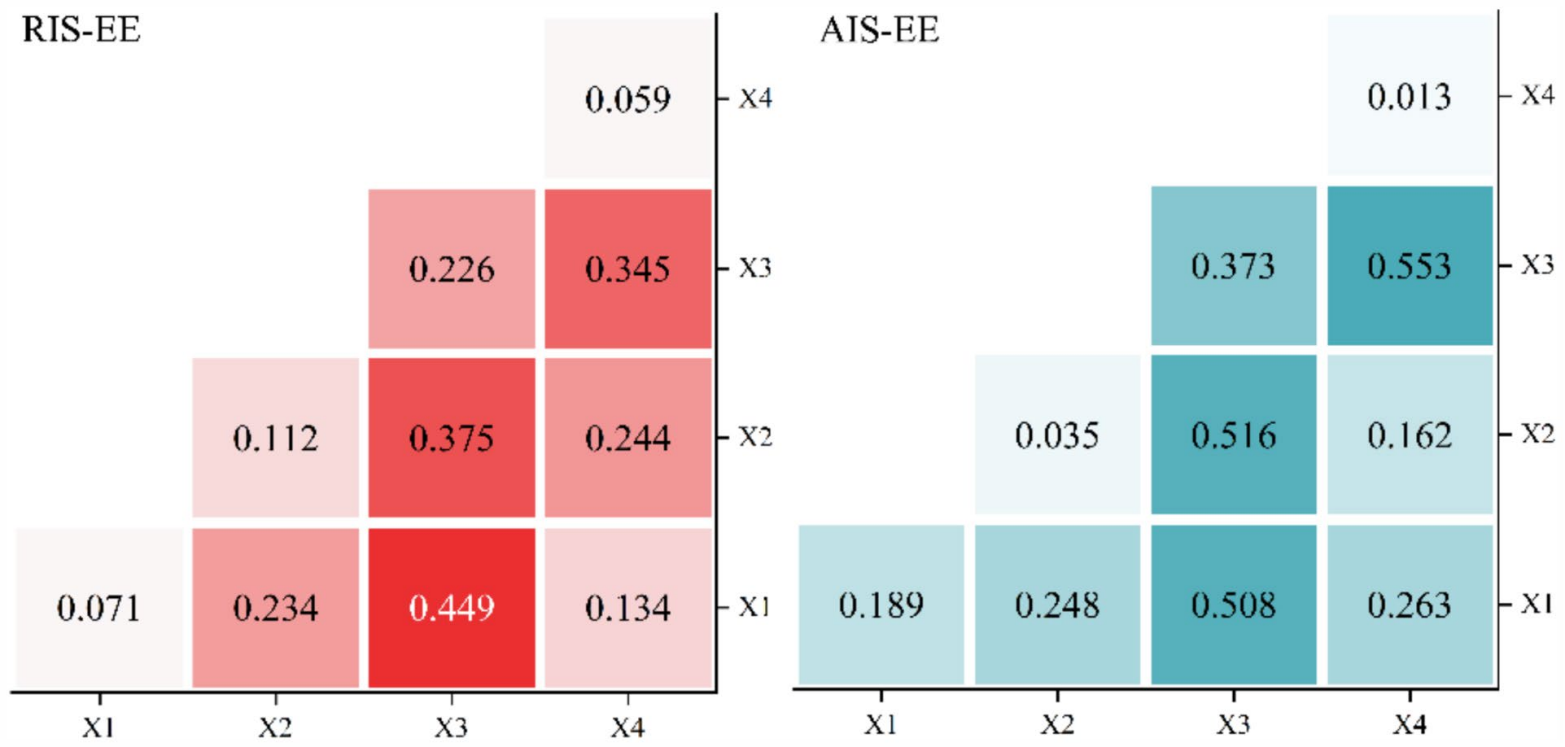
Interaction of multi-factor detection results.

The detection results of variable interaction effects are shown in Figure 13. In the detection of coupling degree of RIS-EE, X1 (economy) and X3 (population) have the strongest interaction effects, which is 0.449. In the coupling detection of AIS-EE, the interaction effect of X2 (communication) and X3 (population) is the strongest, which is 0.516.

##### 5.3.3.2 Result analysis of Moran’I

In order to explore the spatial agglomeration effect of coupling degree between industrial upgrading and ecological environment, we selected the Moran index of AIS-EE to test it, and the results are shown in Table 6. From 2010 to 2021, the Moran index passed the significance test, and the Moran value fluctuates from 0.119 in 2010 to 0.211 in 2021, which proves that there is a spatial agglomeration effect of the coupling degree of AIS-EE.

**Table 6:** Moran index of AIS - EE coupling degree.

Year	Moran’s I	E(I)	sd(I)	z	*p*-value*
2010	0.119	−0.056	0.118	1.48	0.069*
2011	0.107	−0.056	0.12	1.356	0.087*
2012	0.163	−0.056	0.119	1.838	0.033^**^
2013	0.203	−0.056	0.119	2.164	0.015^**^
2014	0.301	−0.056	0.129	2.772	0.003^***^
2015	0.351	−0.056	0.13	3.142	0.001^***^
2016	0.398	−0.056	0.131	3.457	0^***^
2017	0.227	−0.056	0.129	2.188	0.014^**^
2018	0.154	−0.056	0.128	1.642	0.05^**^
2019	0.239	−0.056	0.13	2.257	0.012^**^
2020	0.262	−0.056	0.132	2.41	0.008^***^
2021	0.211	−0.056	0.132	2.021	0.022^**^

**Figure 14:**
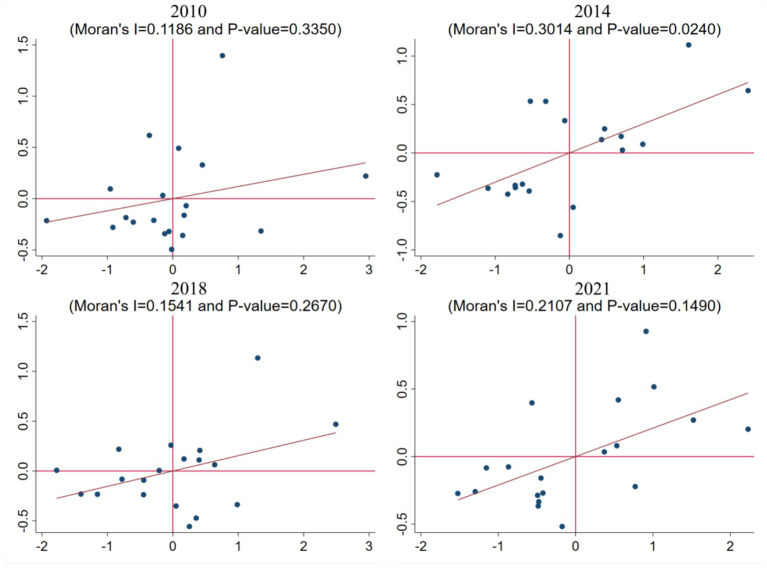
Local Moran scatter plot of AIS-EE coupling degree.

The years 2010, 2014, 2018, and 2021 are selected for the local Moran test, as shown in Figure 14. Moran values of four years are, respectively, 0.1186, 0.3014, 0.1541, 0.2107, and combined with the scatter diagram can be seen, from 2010 to 2021, high concentration and low concentration will increase the amount of data points, proof of AIS - EE coupling is spatial spillover effect remarkable enhancement.

## 6 Discussion and conclusions

### 6.1 Discussion

Based on the above research conclusions, the comprehensive evaluation scores of the ecological environment of the 19 resource-based cities in Northeast China were generally low, with the top-ranked Heihe having an average score of only 0.4939. This indicates that resource-based cities do have environmental damage caused by issues such as resource dependence or irrational industrial structure, and frequent industrial activities can also exacerbate environmental problems, thus putting considerable pressure on the ecological carrying capacity of these cities (26, 67). The gap in the comprehensive ecological environment scores among cities was not significant, but their growth rates varied considerably. For example, the fastest growth was found in Baishan of Jilin, with a cumulative increase of 81.92%. While in Heilongjiang, the ecological environment of three cities had deteriorated: the comprehensive score of Mudanjiang’s ecological environment had decreased by 13.15%, Yichun by 6.09%, and Daqing by 3.66%. The possible reasons were as follows: First, Heilongjiang, which is also located in northeast China, has a higher latitude, cold climate and relatively harsh natural conditions. In addition, the over-exploitation of resources in history has led to the deterioration of the ecological environment in some areas, and even the occurrence of “resource curse” (39). Second, the issue of population loss. The permanent population of Heilongjiang has decreased year by year from the peak of 38.33 million in 2010 to 30.62 million by the end of 2023, a decrease of 20.11% in 13 years. The decline in population will lead to slower growth in the service sector and an increase in the share of industry, further increasing environmental pressure; Third, the government has not paid enough attention to environmental governance and has not invested enough in it (68).

Considering RIS and AIS, the 19 resource-based cities in Northeast China had relatively significant differences in these two aspects. The RIS scores of each city were relatively low, ranging from 0.0260 to 0.4608; while the AIS scores were relatively high, ranging from 0.4904 to 2.5534. This indicates that these cities have attached greater importance to the development of tertiary industry in the past decade or so, but they have not allocated resources in a rational and scientific manner, and have not fundamentally solved the problem of irrational industrial structure in resource-based cities, because the development and improvement of tertiary industry requires the supporting support of primary and secondary industries.

Most existing studies on the industrial structure of resource-based cities primarily focus on three aspects: the impact of environmental regulations on industrial structure upgrading (69), the influence of industrial structure upgrading on energy efficiency and carbon emissions (18, 70), and the interrelationship between industrial structure and natural resources or energy endowments (71). However, these studies often overlook the coupling relationship between industrial structure upgrading and the ecological environment, as well as their synergistic development. To bridge this research gap, this paper builds on existing literature to conduct a detailed analysis. It innovatively subdivides industrial structure upgrading into RIS and AIS. By examining their coupling with ecological environmental systems, this study reveals critical differences and underlying mechanisms of their CCD. The results of the study show that among these 19 cities, the CCD of RIS and EE was low, while the CCD of AIS and EE was high, which indicates that resource-based cities have not done enough in the rational allocation of resources, and also indicates that such a division of industrial structure upgrading is meaningful. Compared with previous studies, this paper transcends the traditional single-directional analyses of industrial structure and environmental regulation or energy utilization. Instead, it establishes a comprehensive research framework that examines the coupling between industrial structure and ecological environment system, which provides a new analytical tool for the green development of resource-oriented cities.

### 6.2 Conclusion

In this study, the CCD of industrial structure upgrading and ecological environment in 19 resource-based cities in Northeast China were calculated and analyzed. The industrial structure upgrading was divided into RIS and AIS, the former was measured by Theil index, the latter was measured by the output value ratio between the tertiary industry and the secondary industry. The ecological environment system considered three levels of ecological environment status, ecological environment pressure and ecological environment protection. Nine indexes were selected, Entropy-CRITIC-TOPSIS model was adopted for comprehensive evaluation, and coupling coordination degree model was used to analyze the coupling coordination of the two systems.

The results showed that the coupling coordination of RIS and EE in these 19 cities was not ideal, and the CCD calculation result of 17 cities was less than 0.4. According to the classification criteria, the proportion of CCD of RIS and EE development into uncoordinated stage was as high as 89.47%, and in comparison, the coupling coordination of AIS and EE in these cities was better. From the perspective of average value, the CCD of AIS and EE in all cities was higher than 0.4, which was basically coordinated or above, and three cities (accounting for 15.79%) even reach moderate coordination. From the perspective of the two dimensions of industrial structure upgrading, the RIS means more reasonable resource allocation, while the AIS means that high-value industries replace backward industries by increasing the unit consumption value (46, 47). According to the research conclusions, resource-based cities in Northeast China should also improve in terms of rational allocation of resources, coordinated development of environmental protection and resource utilization. For example, reduce dependence on a single resource and achieve diversified and intelligent industrial development. Simultaneously, strengthen the awareness of environmental protection, increase investment in environmental protection, adopt clean production technologies, reduce pollution emissions, and protect the ecological environment. In terms of resource utilization, it is necessary to improve resource utilization efficiency, implement a circular economy model, promote the recycling of waste, form a virtuous cycle of resource conservation and environmental friendliness, and ensure the coordinated development of economy, society, and environment.

Although this paper provides a detailed analysis of the coordinated development between industrial structure upgrading and the ecological environment in resource-based cities, it has certain limitation. The study focuses exclusively on 19 resource-based cities in Northeast China, where the climate and regional characteristics may limit the applicability of the results to other areas. Therefore, the findings may not be directly transferable to resource-based cities in other regions. Future research could extend the analysis to resource cities in different regions to further assess the generalizability of the model.

## Data Availability

The data analyzed in this study is subject to the following licenses/restrictions: data for this study are available upon reasonable request from the corresponding authors. Requests to access these datasets should be directed to Yifu Lu, yifu_lu_anu@163.com.
